# Candidate Effectors from *Botryosphaeria dothidea* Suppress Plant Immunity and Contribute to Virulence

**DOI:** 10.3390/ijms22020552

**Published:** 2021-01-07

**Authors:** Chuan-Jie Zhang, Shi-Xing Wang, Yan-Na Liang, Sheng-Hui Wen, Bao-Zhu Dong, Zheng Ding, Li-Yun Guo, Xiao-Qiong Zhu

**Affiliations:** Key Lab of Pest Monitoring and Green Management, College of Plant Protection, China Agricultural University, Beijing 100193, China; zhangchuanjie@cau.edu.cn (C.-J.Z.); lrowangsx@gmail.com (S.-X.W.); ynliang@genedenovo.com (Y.-N.L.); wenshenghui88@gmail.com (S.-H.W.); dongbaozhu2020@imau.edu.cn (B.-Z.D.); zheng.ding@dbn.com.cn (Z.D.); ppguo@cau.edu.cn (L.-Y.G.)

**Keywords:** *Botryosphaeria dothidea*, host–pathogen interaction, plant immunity, effector, virulence

## Abstract

Fungal effectors play important roles in host–pathogen interactions. *Botryosphaeria dothidea* is an ascomycetous fungus that is responsible for the diseases of hundreds of woody plant species, including apple ring rot, which seriously affects apples worldwide. However, little is known about the effectors of *B. dothidea*. In this study, we analyzed the *B. dothidea* genome and predicted 320 candidate effector genes, 124 of which were successfully amplified and cloned. We investigated the effects of these genes on plant cell death in *Nicotiana benthamiana* while using a transient expression system. Twenty-four hours after initial inoculation with *Agrobacterium tumefaciens* cells carrying candidate effectors, the infiltrated leaves were challenged with *A. tumefaciens* cells carrying the *BAX* gene. In total, 116 candidate effectors completely inhibited, while one partially inhibited, the programmed cell death (PCD) of *N. benthamiana* induced by BAX, whereas seven candidate effectors had no effect. We then further tested seven candidate effectors able to suppress BAX-triggered PCD (BT-PCD) and found that they all completely inhibited PCD triggered by the elicitors INF1, MKK1, and NPK1. This result suggests that these effectors were activated in order to suppress pathogen-associated molecular pattern-triggered immunity. The signal peptides of these candidate effectors exhibited secretory activity in yeast (pSUC2 vector). Moreover, the respective deletion of *Bdo_11198* and *Bdo_12090* significantly reduced the virulence of *B. dothidea*. These results suggest that these effectors play important roles in the interaction of *B. dothidea* with its hosts.

## 1. Introduction

*Botryosphaeria dothidea* is a worldwide pathogen that infects woody plants in more than 24 genera, including *Malus* and *Pyrus* [1]. The fungus, which causes fruit rot, leaf spot, twig dieback, stem canker, and tree death, is a constant threat to the commercial forest industry as well as native ecosystems [1,2,3,4,5]. For instance, apple ring rot (white rot) that is caused by *B. dothidea* severely impacts apple production in China, where it typically weakens trees and causes more than 20% fruit loss [4,6,7]. Although genomic data for *B. dothidea* are available [1,8,9], the pathogenesis of *B. dothidea* is still unknown, which is mainly due to the lack of gene disruption protocol for this pathogen. Our recently established gene deletion protocol [10] makes genetic manipulation of this coenocytic woody plant pathogen possible.

During the interaction of plants and pathogens, plants develop two layers of innate immunity in response to pathogen infection [11]. The first layer of immunity, which is referred to as pathogen-associated molecular pattern-triggered immunity (PTI), is triggered by pathogen-associated or microbe-associated molecular patterns (PAMPs or MAMPs). PTI signaling mechanisms are involved in reactive oxygen species production, cytosolic calcium accumulation, callose deposition, the activation of mitogen-activated protein kinase (MAPK) signaling cascades, and changes in defense gene expressions [12,13,14,15]. The adapted pathogen secretes effector proteins into plant cells and it inhibits PTI to achieve infection [16,17,18]. The second layer of immunity, effector-triggered immunity (ETI), has arisen via the evolution of several plant resistance (R) proteins that recognize various effectors [11,19]. In response, some of the pathogens have developed effectors that interfere with ETI [11].

Pathogen-secreted effectors play an important role in the plant–pathogen interface during infection [20]. The systematic screening and identification of effectors inducing or suppressing plant immunity has rapidly progressed for many pathogens (Table S1). *Agrobacterium*-mediated infiltration of tobacco (*Nicotiana benthamiana*) has been widely used in order to identify candidate effector genes of various pathogens. In an *A. tumefaciens*-mediated transient expression assay of *N. benthamiana*, for example, a large majority of tested RXLR (as defined by their conserved N-terminal arginine, any amino acid (aa), leucine, arginine motif) effectors from the oomycete *Phytophthora sojae* were able to suppress programmed cell death (PCD) that is triggered by the mouse protein BAX (BT-PCD) [21]. Various studies have confirmed that the *Ph. sojae* effector Avh240 can inhibit host aspartic protease secretion in order to promote infection, the RXLR effector Avh238 can destabilize soybean Type2 GmACSs to suppress ethylene biosynthesis and promote infection, and the *Ph. sojae* effector Avr1d can inhibit the ubiquitination activity of GmPUB13 to facilitate infection [22,23,24]. Seventeen PvRxLR effector candidates from another oomycete, *Plasmopara viticola*, completely suppressed PCD that is elicited by BAX, INF1, PsCRN63, PsojNIP, PvRxLR16, and R3a/Avr3a [25], while the effector PvRXLR131 was able to suppress plant immunity by targeting plant receptor-like kinase inhibitor BKI1 [26]. Among fungi, five effectors from *Pyricularia oryzae* (syn. *Magnaporthe oryzae*) were found to induce host cell death in protoplasts in a transient expression system [27]. In another case, some *P. oryzae* candidate effectors that suppressed BT-PCD in *N. benthamiana* leaves were found to be involved in fungal propagation and pathogenicity [28]. Thirteen putative effectors from *Ustilaginoidea virens* caused necrosis or necrosis-like phenotypes in *N. benthamiana* [29]. In addition, *U. virens* effector SCRE1 could suppress rice immunity via a small peptide region [30]. In the woody plant pathogen *Valsa mali*, the VmHEP1 and VmHEP2 effectors were able to suppress PCD elicited by BAX in *N. benthamiana* leaves, and the double deletion of *VmHEP1* and *VmHEP2* notably attenuated *V. mali* virulence in both apple twigs and leaves [31]. In nematodes, 78 putative effectors of *Heterodera avenae* were found to suppress BT-PCD and seven caused cell death in *N. benthamiana* [32]. In addition, GLAND5 was determined to interact with the pyruvate dehydrogenase subunit of plants in order to promote nematode parasitism [33].

As a hemi-biotrophic fungal pathogen, *B. dothidea* attacks hundreds of woody plants; however, little is known regarding the pathogenic mechanism of *B. dothidea.* Functional analysis of effectors of *B. dothidea* should help to elucidate the interaction between *B. dothidea* and its hosts. In this study, we predicted candidate effectors of *B. dothidea* by analyzing the genome of *B. dothidea* ZY7 and investigated their effects on plant immunity. Most of the candidate effectors were able to suppress PCD triggered by BAX, according to our results. Finally, we disrupted two candidate effector genes and compared the pathogenicities of the resulting gene deletion transformants and the wild type.

## 2. Results

### 2.1. Bioinformatic Prediction of the B. dothidea Effectome

**Figure 1:**
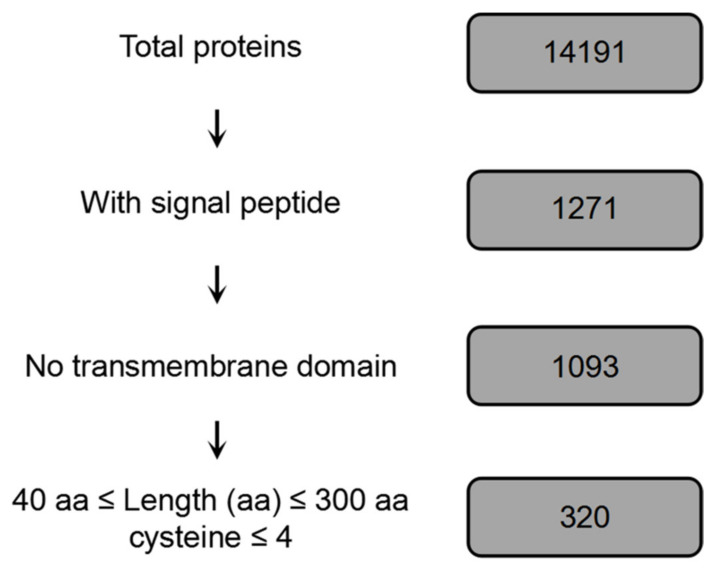
Pipeline for *B. dothidea* candidate effector proteins prediction.

A series of domain and protein structure analyses of *B. dothidea* ZY7 genomic data (14,191 genes; unpublished data) were used in order to predict the *B. dothidea* effectome [34]. In total, 1,271 proteins carrying N-terminal signal peptides (SPs), accounting for approximately 8.6% of all putative genes in the genome, were identified while using SignalP 4.0 and WolfPSORT. We obtained 320 candidate effectors of *B. dothidea* after excluding proteins whose predicted transmembrane domains (TMDs) overlapped with SPs (Figure 1).

Analyses of candidate effector revealed that 119 of the 320 genes were functionally annotated proteins (Table S2). For example, eight candidate effectors were found to contain a glycoside hydrolase motif, while five candidate effectors included a pectate lyase domain, and one candidate effector possessed a cell wall integrity and stress response component (Table S2). Uncharacterized proteins were the remaining candidate effectors.

### 2.2. Suppression of BT-PCD by a Majority of B. dothidea Candidate Effectors

**Figure 2:**
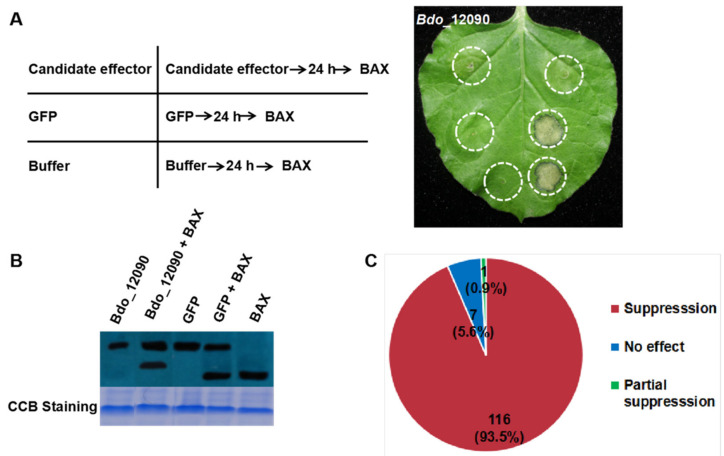
Suppression of BAX-triggered programmed cell death (BT-PCD) in *N. benthamiana* by candidate effectors in *B. dothidea* (example Bdo_12090). (**A**) Cell death suppression of Bdo_12090 by *Agrobacterium*-mediated transient expression in *N. benthamiana*. *N. benthamiana* leaves were infiltrated with buffer and *Agrobacterium* cells carrying *Bdo_12090* or the *GFP* gene; infiltration was either performed alone or followed 24 h later by infiltration with *Agrobacterium* cells carrying a mouse *Bax* gene. The Representative photo was acquired 5 d after the last infiltration. Positions of Candidate effector, GFP, and Buffer in the left table correspond to the infiltration sites on the right leaf photograph, respectively. (**B**) Western blotting was used to confirm the expression of Bdo_12090, GFP and BAX in (**A**), and equal loading is indicated by coomassie blue (CBB) staining. (**C**) The number of candidate effectors and their proportion that showed suppression or no effect of BT-PCD on leaves of *N. benthamiana*.

In order to investigate the function of the 320 candidate effectors of *B. dothidea*, we amplified their encoding genes. A total of 124 predicted effector genes were successfully amplified by Polymerase Chain Reaction (PCR) and then sub-cloned into the PVX vector pGR107. We investigated the ability of these effector genes to induce PCD or suppress BT-PCD through *Agrobacterium*-mediated transient expression in *N. benthamiana*. As an example, Figure 2A presents the results that were obtained using Bdo_12090. In this case, leaves infiltrated with BAX protein after the introduction of Bdo_12090 showed no evidence of PCD, whereas those that were infiltrated with GFP or buffer experienced PCD (Figure 2A). Western blot analysis revealed the translational levels of the candidate effector, BAX and GFP in *N. benthamiana* leaves after infiltration. The level of BAX protein in tissues showing suppression was identical to that of leaves with no evidence of suppression, as illustrated in Figure 2B. This result suggests that Bdo_12090 can completely suppress BT-PCD.

Among the 124 tested candidate effectors, 116 (93.5%) completely suppressed BT-PCD, while one (0.9%) partially suppressed BT-PCD, and the remaining seven (5.6%) had no obvious effect (Figure 2C). These results suggest that 117 of the candidate effectors can interfere with plant defense response.

### 2.3. Functional Validation of Predicted SPs of Candidate Effectors

**Figure 3:**
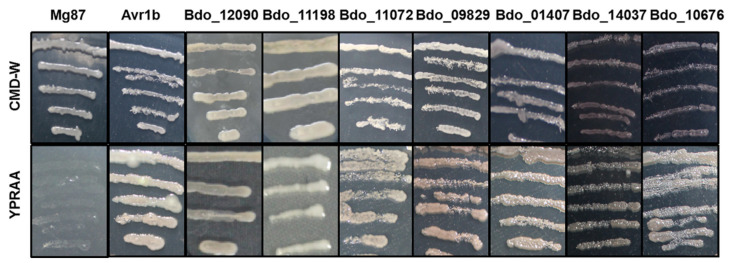
Functional validation of signal peptides of candidate effectors from *B. dothidea* using a yeast invertase secretion assay. The transformed yeast strains of YTK12 were able to grow on YPRAA media with raffinose as sole carbon source. The N-terminal sequences of *Magnaporthe oryzae* Mg87 and *Phytophthora sojae* Avr1b were used as negative and positive controls, respectively.

We used the yeast secretion system for validation in order to analyze the secretory activity of the predicted SPs of candidate effectors [29,35]. We randomly selected seven candidate effectors that suppressed the BT-PCD to test. Similar to the positive control Avr1b, the predicted SPs of the seven candidate effectors restored the growth of invertase-deficient yeast on YPRAA medium (Figure 3). This result suggests that the N-terminal peptides of these seven proteins are able to guide the secretion of the truncated invertase and the predicted SPs of the seven candidate effectors, thus, appear to be functional.

### 2.4. Candidate Effector Suppression of PCD Triggered by Different Elicitors

**Figure 4:**
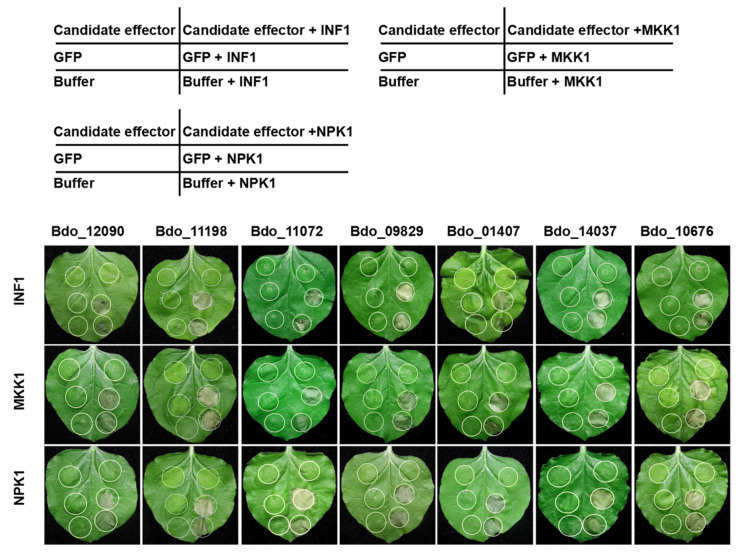
Transient expression of candidate effectors in *N. benthamiana* suppressed programmed cell death triggered by INF1, and two resistance-related mitogen-activated protein kinases (MAPKs) (MKK1 and NPK1). *N. benthamiana* leaves were infiltrated with *Agrobacterium tumefaciens* cells containing candidate effectors, GFP or Buffer (as a control), either alone or followed 24 h later by infiltration with *Agrobacterium cells* carrying PVX:INF1/MKK1/NPK1. Positions of Candidate effector, GFP, and Buffer in the table correspond to the infiltration sites on the below leaf photograph, respectively. 2.5. Involvement of Bdo_11198 and Bdo_12090 in the Pathogenicity of *B. dothidea*.

Plant pathogenic microorganisms can suppress plant PTI [11]. PAMP INF1 of *Phytophthora infestans* is a well-characterized plant immunity elicitor that can trigger PCD; in addition, the overexpression of MKK1 and NPK1 of *N. benthamiana*, important components of the mitogen-activated protein kinases (MAPK) cascades of PTI, also trigger PCD in *N. benthamiana* [36,37,38,39]. We further tested whether the above seven candidate effectors can suppress PCD that is triggered by INF1, MKK1, or NPK1. Interestingly, all seven exhibited the ability to suppress PCD that is induced by the three different elicitors (Figure 4). This result indicates that these candidate effectors of *B. dothidea* can suppress PCD that is associated with PTI response.

**Figure 5:**
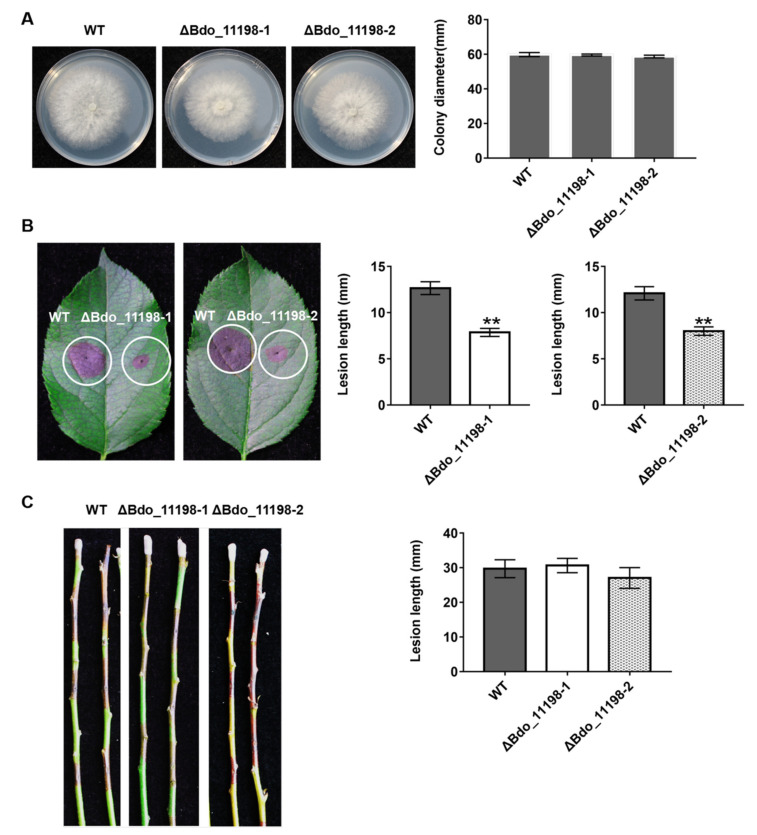
Functional analysis of effector Bdo_11198 in *B. dothidea* determined by colony characteristics and pathogenicity of gene knockout transformants. (**A**) The colony diamater of Bdo_11198 knockout mutants and wild-type (WT) on potato dextrose agar (PDA). (**B**) Pathogenicity on wounded apple leaves and (**C**) on wounded shoots were tested by inoculating with mycelial blocks of WT and Bdo_11198 knockout mutants, respectively. The lesion size was measured and symptoms were photographed five days after inoculation. Data represent means ± standard error of mean (SEM) from two independent experiments. **: significant at *p* < 0.01.

**Figure 6:**
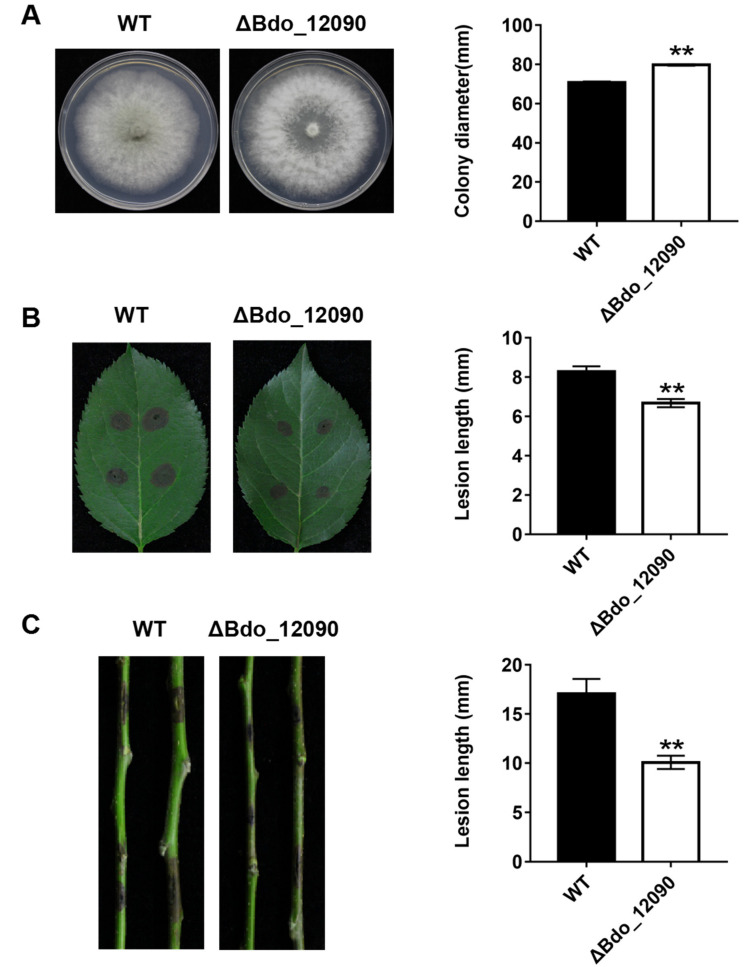
Functional analysis of effector Bdo_12090 in *B. dothidea* determined by colony characteristics and pathogenicity of gene knockout transformants. (**A**) The colony diameter of Bdo_12090 knockout mutants and WT on PDA. (**B**) Pathogenicity on wounded apple leaves and (**C**) on wounded shoots were tested by inoculating with mycelial blocks of WT and Bdo_12090 knockout mutants, respectively. Lesion size was measured and symptoms were photographed five days after inoculation. Data represent means ± standard error of mean (SEM) from two independent experiments. **: significant at *p* < 0.01.

We deleted two representative genes in order to investigate the function of the candidate effectors identified in this study: Bdo_11198, which encodes an uncharacterized protein, and Bdo_12090, which has a CFEM domain containing eight cysteines. We successfully obtained two *Bdo_11198* deletion mutants (Figure S1) and one *Bdo_12090* deletion mutant (Figure S2). The *Bdo_11198* deletion mutants had similar colony morphologies and mycelial growth rates on PDA plates as the wild-type strain (Figure 5A). When compared with the wild type, these two mutants induced significantly smaller lesions on wounded apple leaves (Figure 5B), but similar ones on apple shoots (Figure 5C). Interestingly, the *Bdo_12090* deletion mutant had a much faster mycelial growth rate on PDA plates when compared with the wild-type strain (Figure 6A); however, it induced significantly smaller lesions on both wounded apple leaves and shoots as compared with the wild type (Figure 6B,C). These results indicate that the candidate effectors Bdo_11198 and Bdo_12090 are involved in the pathogenicity of *B. dothidea*.

## 3. Discussion

Pathogen-secreted effector proteins play important roles in plant–pathogen interactions [40,41,42,43,44,45], but little is known regarding the effectors in *B. dothidea*. In this study, we predicted 320 candidate effectors that are based on bioinformatics analyses of the *B. dothidea* genome. We successfully amplified 124 of these effectors and investigated their ability to suppress BT-PCD in *N. benthamiana* following *Agrobacterium*-mediated infiltration. We determined that 116 effectors were able to completely suppress BT-PCD in *N. benthamiana*, with one additional effector causing partial suppression (Figure 2 and Table S2). In addition, seven randomly selected representative candidate effectors were able to suppress PCD that is triggered by MKK1, NPK1, and INF1 in *N. benthamiana*, and the secretory activity of their SPs was confirmed through a yeast secretion assay. Moreover, a pathogenicity assay of two *B. dothidea* gene deletion transformants revealed that Bdo_11198 and Bdo_12090 are pathogenicity-related genes.

The *Agrobacterium*-mediated transient expression assay in *N. benthamiana* is a model system that has been used in order to identify effectors in oomycetes, fungi, and nematodes [21,32,46]. We also used this non-host model system to screen candidate effectors in *B. dothidea.* Some previous studies have shown that plant pathogens effectors identified in non-hosts have been discovered to play important roles in host plants. For example, *Ph. sojae* RXLR effectors Avh172 and Avh6 have been shown to suppress ETI in both non-host *N. benthamiana* and in host soybean [21]. *Puccinia striiformis* f. sp. *tritici* effectors PstGSRE1, Pst_12806, and Pst18363 are able to suppress PCD in both *N. benthamiana* and wheat [47,48,49].

In this study, 320 candidate effectors in the *B. dothidea* genome were predicted by bioinformatics analysis, but only 124 candidate effector genes were successfully cloned. This low amplification rate may be due to the weak expression of these genes during mycelial growth. It is likely that some candidate effector genes expressed only upon encountering with host plant or under specific conditions. Furthermore, we did not find any candidate effectors that could induce PCD in *N. benthamiana* in our assay. In some previous studies, a large number of candidate effectors were able to suppress PCD, but only a few could induce PCD in *N. benthamiana.* For example, 22 of 23 PvRxLR effector candidates that were identified in *Pl. viticola* [25], seven of 70 candidate effectors from *V. mali* [46], and 25 of 31 candidate effectors from *Uromyces appendiculatus* [50] could suppress BT-PCD, while eight of 27 candidate effectors of *Lasiodiplodia theobromae* were able to suppress PCD in *N. benthamiana* [9]. Interestingly, none of the candidate effector genes in the above-cited studies were able to induce PCD in *N. benthamiana*, which may be because not enough candidate effectors were screened. Besides, it is likely related to the biology of the *B. dothidea,* which has a prolonged latent period or an endophytic phase in host plant [1]. Lacking effectors that induce PCD is favorable for the endophytic stage of a microbe. According to our results, many candidate effectors from *B. dothidea* can suppress plant immunity; this suggests that *B. dothidea* secreted a large number of effectors that play important roles in interaction with its hosts.

In a previous study, we observed that Bdo_11198 suppressed the BT-PCD of *N. benthamiana*; its SP had secretory activity, and this gene was significantly up-regulated during the infection of apple fruit [51]. Moreover, the infiltration with Bdo_11198 significantly increased the infection rate of *Phytophthora nicotianae* in *N. benthamiana* and decreased the accumulation of reactive oxygen species [51]. In the present study, we separately deleted *Bdo_11198* and *Bdo_12090*, which is predicted to have a CEFM domain, from wild-type strain HTLW03 (Figures S1 and S2). Virulence assays revealed that the *Bdo_11198* knockout transformants were significantly compromised in their ability to infect wounded apple leaves, but not wounded shoots, when compared with the wild type (Figure 5). In contrast, the *Bdo_12090* deletion mutant had significantly decreased pathogenicity on both wounded apple shoots and leaves (Figure 6). These results indicate that the effectors of *B. dothidea* contribute to the full virulence in the host and suggest that *Bdo_11198* has various roles in the pathogenicity of the fungus on different tissues of apple. Our results provide valuable information for analyzing *B. dothidea* pathogenesis in apple and other woody plants.

## 4. Materials and Methods

### 4.1. Fungi and Plants

The *B. dothidea* isolate ZY7 (that was isolated from apple in Henan Province, China) was used for screening candidate effectors. Another isolate, HTLW03 (obtained from Chinese flowering crabapple in Shandong Province, China), was used for gene knockout. The wild-type isolates as well as transformants that were generated in this study were cultured on potato dextrose agar (PDA; 200 g potato, 20 g dextrose, 15 g agar, and 1 L water) at 25 °C in darkness. For DNA and RNA extraction, mycelia were collected from cultures that were grown on cellophane on PDA for four to five days. *Nicotiana benthamiana* plants were grown in a growth room for four to six weeks at approximately 25 °C under a 16-h light/8-h dark cycle.

### 4.2. Bioinformatic Analysis and Candidate Effector Prediction

Candidate effector genes of *B. dothidea* with the following characteristics were predicted, as previously described [52]: (1) encoding a protein with a length of less than 300 amino acids and containing (2) a signal peptide (SP) sequence, (3) no transmembrane domain sequence, and (4) more than four cysteine residues. Figure 1 shows the procedure used for effector prediction of *B. dothidea*. Total proteins were predicted based on the genome sequence of *B. dothidea* strain ZY7 (unpublished data) [34]. SPs of these proteins were predicted using SignalP 4.0 with default parameters. The well-validated software WolfPSORT was used to predict the subcellular localization of proteins. Protein transmembrane domains (TMDs) were predicted using TMHMM. Proteins whose predicted TMDs overlapped with their SPs were excluded, as these proteins were likely to be retained in the plasma membrane.

### 4.3. Construction of A. tumefaciens Binary PVX Vectors

The total RNA was extracted from mycelium of *B. dothidea* isolate ZY7 while using an RNA isolation kit (Omega Bio-Tek, Norcross, GA, USA). First-strand cDNA was synthesized from the extracted RNA using a Reverse Transcriptase M-MLV (RNaseH-) kit (Takara, Dalian, China). The open reading frame (ORF) sequences of candidate effectors (without SP), which were amplified from the cDNA of ZY7, and GFP were respectively inserted into the *Potato Virus X* (PVX) vector pGR107 [53] with a 3× flag-tag fused at the N-terminus by the method of digestion and connection using a ClonExpress II One Step Cloning kit (Vazyme Biotech, Nanjing, China). Table S3 lists the primers used for vector construction. After verification through sequencing, the generated construct was transformed into *A. tumefaciens* strain GV3101 by electroporation.

### 4.4. Cell Death Induction/Suppression Assay in N. benthamiana

The assays of *A. tumefaciens*-mediated transient gene expression in *N. benthamiana* were performed, as previously described [21]. Specifically, the cells of *A. tumefaciens* carrying candidate effectors were grown overnight in Luria–Bertani medium containing 50 mg/mL kanamycin in a shaker at 28 °C and 180 rpm. The *A. tumefaciens* cells were harvested, washed three times with sterile double-distilled H_2_O, and then re-suspended in infiltration buffer (10 mM MES [pH 5.7], 10 mM MgCl_2_, and 150 µM acetosyringone) to a final OD_600_ of 0.5. After standing at room temperature for 3 h, the *A. tumefaciens* cells carrying candidate effectors were initially infiltrated via needleless syringes into the leaves of 4–6-week-old *N. benthamiana* plants. Fifteen leaves, three from each tobacco plant, were used per gene. Infiltrations of buffer and *A. tumefaciens* cells carrying pGR107-GFP were used as the controls. The leaves were initially infiltrated and then, 24 h later, the same infiltration site was challenged with *A. tumefaciens* cells carrying BAX, INFI, MKK1, or NPK1. The entire assay was repeated. Cell death symptoms on infiltrated leaves were observed and photographed six days after initial infiltration.

### 4.5. Western Blotting

*Nicotiana benthamiana* leaves were harvested three days post-infiltration. The protein extracts were prepared by grinding 100 mg of leaf tissue in extraction buffer (220 mM Tris-HCl [pH 7.4], 25 mM sucrose, 1 mM MgCl_2_, 50 mM KCl, and 0.5% β-mercaptoethanol). The presence of tested proteins in *N. benthamiana* was confirmed by Western blot analysis while using monoclonal anti-Flag M2 antibody solution (1:5000; Sigma, Saint Louis, MO, USA) as the primary antibody, and a horseradish peroxidase tag-conjugated goat anti-Mouse IgG as the secondary antibody (1:5000; CWBIO, Cambridge, MA, USA). The results were visualized with an electrochemiluminescence detection system (Bio-Rad, Hercules, CA, USA) [29].

### 4.6. Validation of the SP Secretory Activities of Candidate Effectors

The functional validation of predicted SPs of candidate effectors was conducted while using a yeast secretion assay, as previously reported [35]. The predicted SP sequences were amplified using the primers that are listed in Table S4 and then the predicted SP sequence of each candidate effector was fused with a truncated SUC2 gene that encodes an invertase without a SP using a ClonExpress II One Step Cloning kit (Vazyme Biotech, Nanjing, China). The resulting constructs were verified by sequencing and subsequently transformed into invertase-deficient yeast strain YTK12 while using a Frozen-EZ yeast transformation II kit (Zymo Research, Irvine, CA, USA), as previously described [29]. Transformants were selected on yeast minimal tryptophan dropout medium (CMD-Wmedium, 0.67% yeast N base without amino acids, 0.075% tryptophan dropout supplement, 2% sucrose, 0.1% glucose, and 2% agar). The yeast colonies were replica-plated onto YPRAA plates (1% yeast extract, 2% peptone, 2% raffinose, and 2 µg of antimycin A per liter) for invertase secretion assays. The secretion signal of *P*. sojae Avr1b leading to the secretion of the invertase was used as a positive control, and the N-terminal SP of Mg87 in *P*. oryzae, used as the negative control.

### 4.7. Generation of Gene Deletion Transformants

For target gene deletion, polyethylene glycol (PEG)-mediated homologous-recombination gene deletion was performed and verified while using previously described strategies [10]. We generated a gene homologous recombination (GHR) plasmid containing a hygromycin-resistance gene (*Hph*) with flanking sequences of the target gene. The 5′- and 3′-flanking regions (approximately 1000 bp each) of the target gene were amplified and then cloned into a pMD19-T vector at sites that were located upstream and downstream of *Hph*, respectively (Figures S1 and S2).

A cassette, including the 5′- and 3′-flanking sequences of the target gene, was amplified from the GHR plasmid and then transformed into the protoplasts of HTLW03 by the PEG-mediated transformation method, and the gene deletion transformants were screened, as described by Dong and Guo [10]. The gene deletion mutants were verified by PCR while using the primer pairs that are listed in Table S5.

### 4.8. Morphology and Pathogenicity Assay

The WT strain HTLW03 and its transformants were grown on PDA medium, with three replicated plates per strain. After two days incubation, the colony characteristics were examined, and the diameter of the colony was measured. A pathogenicity test was conducted using a previously described method [4], with some modifications. Shoots and detached leaves of apple (*Malus domestica* Borkh. ‘Fuji’) were surface sterilized with 70% ethanol and then rinsed three times with sterilized distilled water. Each shoot was wounded with a nail (2 mm in diameter and 3 mm in depth), and the leaves were wounded with a sterilized needle. A mycelial plug (5 mm in diameter), which was cut from the edge of a two-day-old colony, was then placed on the wounded site. Inoculated leaves were placed in a Petri dish lined with filter paper, while the inoculated shoots were placed in a plastic box lined with filter paper. Both types of plates containing sterilized water at a shallow depth maintained high humidity and were then incubated at 25 °C. Five shoots (approximately 20 cm long, with three or four inoculation sites per shoot) and leaves were used for each tested isolate. The lesion diameters were measured three to five days after inoculation. All of the experiments were repeated.

### 4.9. Statistical Analyses

The statistical significance of differences (*p* = 0.05) between the treatments and controls was assessed using Student’s *t*-test in SPSS v18.0.

## Data Availability

The data presented in this study are available in the article.

## Supplementary Materials

The following are available online at https://www.mdpi.com/1422-0067/22/2/552/s1, Figure S1: Schematic diagram of gene disruption of Bdo_11198 and PCR verification of Bdo_11198 deleted transformants, Figure S2: Schematic diagram of gene disruption of Bdo_12090 and PCR verification of Bdo_12090 deleted transformants. Table S1: Identifying candidate effector genes in various pathogens by using agrobacterium-mediated infiltration of tobacco (*Nicotiana benthamiana*), Table S2: Suppression effect of BT-PCD and putative function of candidate effectors from *B. dothidea*, Table S3: List of oligonucleotide primers used for amplifying candidate effector genes in this study, Table S4: Primers used for amplifying signal peptide of candidate effector, Table S5: Primers used for gene knockout.
