# Supplementary material for: Candidate Effectors from Botryosphaeria dothidea Suppress Plant Immunity and Contribute to Virulence
Source: Int J Mol Sci. 2021 Jan 7;22(2):552. doi: 10.3390/ijms22020552 (PMC7826910; doi:10.3390/ijms22020552)
Supplement: Supplementary file 1 [file ijms-22-00552-s001.zip › Supplementary Figure S1-S2.pdf]

Supplementary materials

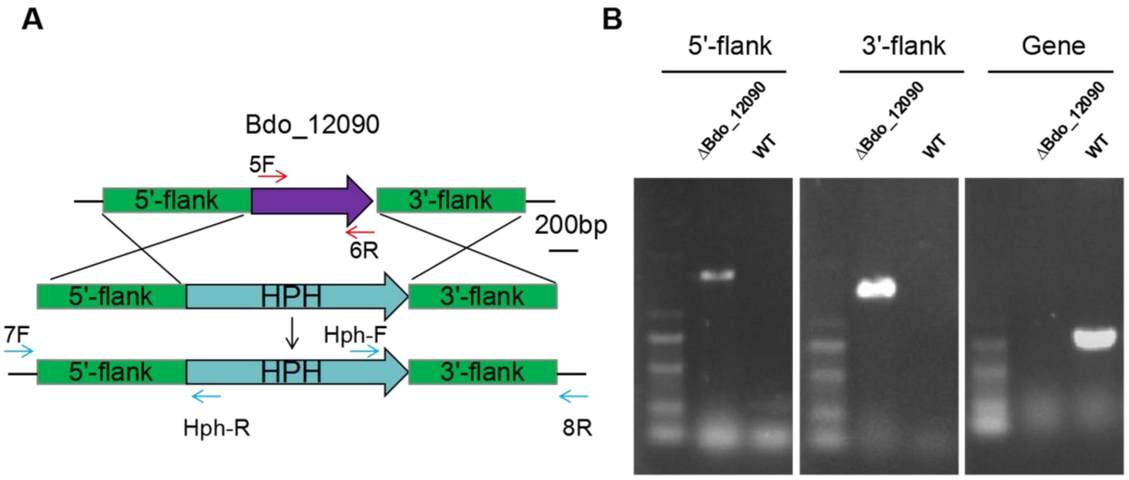

Figure S1. Schematic diagram of gene disruption of *Bdo_11198* and PCR verification of *Bdo_11198* deleted transformants. A. Schematic diagram of gene disruption of *Bdo_11198* gene in wild type strain HTLW03. Orientation of the target gene and Hph are indicated by yellow and light blue arrows, respectively. Upstream (5′) and downstream (3′) flanks of target gene are shown in green color. **B.** knockout mutants was confirmed by genomic PCR analysis.

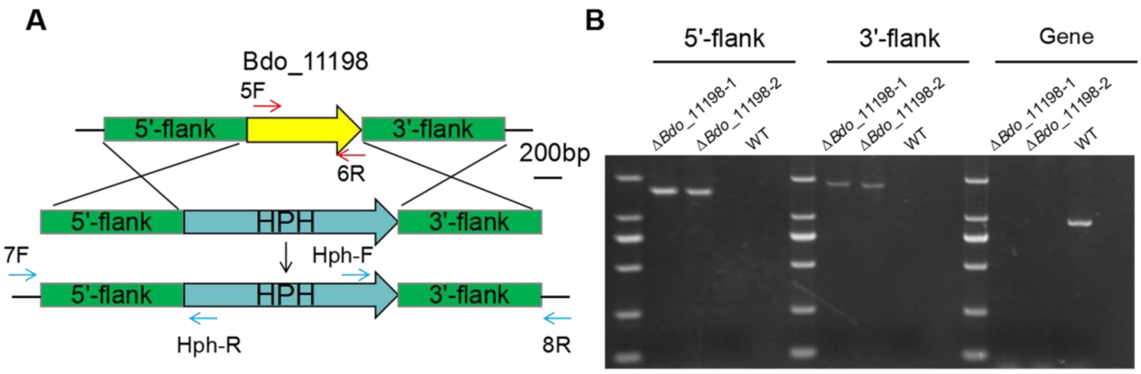

Figure S2. Schematic diagram of gene disruption of *Bdo_12090* and PCR verification of *Bdo_12090* deleted transformants. A. Schematic diagram of gene disruption of *Bdo_12090* gene in wild type strain HTLW03. Orientation of the target gene and Hph are indicated by yellow and light blue arrows, respectively. Upstream (5′) and downstream (3′) flanks of target gene are shown in green color. **B.** knockout mutants was confirmed by genomic PCR analysis.
