# Supplementary material for: Candidate Effectors from Botryosphaeria dothidea Suppress Plant Immunity and Contribute to Virulence
Source: Int J Mol Sci. 2021 Jan 7;22(2):552. doi: 10.3390/ijms22020552 (PMC7826910; doi:10.3390/ijms22020552)
Supplement: Supplementary file 1 [file ijms-22-00552-s001.zip › Supplementary Table S1.pdf]

Table S1. Identifying candidate effector genes in various pathogens by using agrobacterium-mediated infiltration of tobacco (*Nicotiana benthamiana*).

Effectors	Species	Taxonomy	Refence	Annotion
Avh240	*Phytophthora sojae*	Oomycete	[22] (Guo et al., 2019)	Can inhibit host aspartic protease secretion to promote infection
Avh238	*Phytophthora sojae*		[23] (Yang et al., 2019)	Destabilize soybean Type2 GmACSs to suppress ethylene biosynthesis and promote infection
Avr1d	*Phytophthora sojae*		[24] (Lin et al., 2020)	Inhibit the ubiquitination activity of GmPUB13 to facilitate infection
PvRXLR131	*Plasmopara viticola*		[26] (Lan et al., 2019)	Suppress plant immunity by targeting plant receptor-like kinase inhibitor BKI1
SCRE1	*Ustilaginoidea virens*	Fungi	[30] (Zhang et al., 2020)	Could suppress rice immunity via a small peptide region
VmHEP1<br>VmHEP2	*Valsa mali*<br>*Valsa mali*		[31] (Li et al., 2015)	The double-deletion of VmHEP1 and VmHEP2 notably attenuated V. mali virulence in both apple twigs and leaves
GLAND5	*Heterodera avenae*	Nematodes	[33] (Yang et al., 2019b)	Interact with the pyruvate dehydrogenase subunit of plants to promote nematode parasitism
